# The Productivity and Characteristics of Iranian Biomedical Journal (IBJ): A Scientometric Analysis

**DOI:** 10.29252/.22.6.362

**Published:** 2018-11

**Authors:** Hassan Asadi, Ehsan Mostafavi

**Affiliations:** 1 Deputy of Research, Technology and Education, Research Section, Pasteur Institute of Iran, Tehran, Iran; 2 Department of Epidemiology and Biostatistics, Research Centre for Emerging and Reemerging Infectious Diseases, Pasteur Institute of Iran, Tehran, Iran

**Keywords:** Bibliometric analysis, Bibliometrics, Publications

## Abstract

**Background:**

Scientometrics is a discipline that analyzes scientific publications to explore the structure and growth of science. In this work, the quantitative evaluation of the productivity of the Iranian Biomedical Journal (IBJ) is reviewed.

**Methods:**

The analysis was done based on a cross-sectional descriptive and an analytical scientometric study. Data were collected from PubMed, Scopus, and Scimago Databases (2000-2017). Scopus and Scimago were used for data search and feature analysis. Analyzed scientometric indicators included the number of citations, publications, CiteScore, SJR (Scimago Journal Rank), SNIP (source normalized impact per paper), self-citation, and Q (quartile) trend.

**Results:**

The evaluation of 586 documents, published in IBJ from 2000 to 2017, revealed that most of these documents (99.7%) have been published in the areas of biochemistry, genetics, and molecular biology, which yielded to an upgrade in Journal Q ranking from Q4 (in 2000) to Q2 (in 2016).

**Conclusion:**

Nearly all of the scientometric indicators, evaluated in this study, were found on the rise. Therefore, a growing trend from Q2 to Q1 is predicted for the near future. It is recommended that the journal focuses on a specific subject area to improve the indicators and quality of the journal, in a timely manner.

## INTRODUCTION

Scientific journals play central roles in the scientific community[1]. In assessment of scientific performance, bibliometric and citation indicators are among the most important impact measures of scientific literature[2]. Routine evaluation of scientific activities of these journals will provide a clear view of journal motion track and the subject areas[3]. This evaluation is also important for policymakers and their decision making. Scientometrics is one of the sciences that is used by many researchers in various fields to analyze and measure scientific production using different indicators. Major research issues include the measurement of impact and the reference sets of articles to investigate the impact of journals and institutes, understanding of scientific citations, mapping scientific fields, and the production of indicators for use in policy making and management contexts[4,5].

In this study, a scientometric analysis of Iranian Biomedical Journal (IBJ) was carried out. IBJ is an international bimonthly journal, founded in 1996, and continuously being published since October 1997[6]. IBJ covers a wide range of disciplines in the fields of biomedicine and laboratory-based experimental biomedical sciences, viz all aspects of biochemistry, genetics, immunology, cell biology, developmental biology, microbiology, molecular biology, pharmacology, and physiology. IBJ is now being indexed by BIOSIS, PubMed, MEDLINE, Scopus, BIOBASE, Embase, Google Scholar, IranMedex, ISC, Magiran, and Scientific Information Database (SID)[7,8]. IBJ is the publication of Pasteur Institute of Iran (PII), a member of Institute Pasteur International Network, which includes 33 research institutes around the world, and a network of research and expertise to fight against infectious diseases[9].

The objectives of this study were to categorize IBJ documents, based on discipline, year of publication, country territory etc. and to evaluate the pertaining scientometric indices such as CiteScore, SJR (Scimago Journal Rank), SNIP (source normalized impact per paper), citation, self-citation, and total citation.

## MATERIALS AND METHODS

In this paper, attempts have been made to explore IBJ from 2000 to 2017 (an 18-year period), using scientometric indicators, including the number of citations, number of publications, CiteScore, SJR, SNIP, self-citation, Q (quartile trend, distribution of contributions, documents (by authors, year, type, and subject area), and citation. This study covers 586 articles of 33 published issues, during this time period. MS-Excel software was used for data analysis. Both Scopus and Scimago databases were employed for data retrieval and analyses.

CiteScore is defined as the number of citations received by a journal in the last three years divided by the number of documents indexed in Scopus, during those three years. As an example, the calculation of the CiteScore for year 2016 is done based on the following formula:


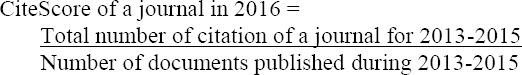


CiteScore is a standard yardstick that provides comprehensive, transparent, and current view of a journal’s impact and will guide journal directors in their policy making[10]. CiteScore allows to monitor the journal’s performance throughout the year, lessening the need to wait until mid-year to see how a journal has performed, during the previous year[11].

SNIP is known as an indicator of the citation impact of scientific journals[12] and using a source-normalized approach corrects differences in citation practices between scientific fields[4,13]. The scientometric indices (such as citation impact, CiteScore, and SNIP) of a journal can be extracted from the Scopus Database. SJR is a prestige metric based on the idea that ’all citations are not created equal[14]. Every scientific document may receive citations from a wide array of journals. To take into account the prestige of the citing journal, in addition to the number of citations, SJR comes to play. The SJR, therefore, is an indication of quality as well as quantity of the visibility of a certain document.

To extract information such as subject area, documents type etc., we have used the “source” of the Scopus Database. Also, by choosing the name of the journal, we chose each of the indicators for this 18-year period. Q rankings are therefore derived from SJR. Based on SJR, journals are ranked and divided into four subject categories. Q1 denotes the top 25%, Q2 between top 50% and top 25%, Q3 top 75% to top 50%, and Q4 bottom 25% of impact factor distribution).

### Year of publication

Table 1 depicts the number of documents published in IBJ per year. Overall, 586 research articles were published with an average of 33 articles per year. The maximum numbers of documents/year (n = 56) occurred in 2017, as opposed to the minimum (n = 16) in 2000.

**Table 1:** Year-wise distribution of papers published in IBJ

Year	No. of volume	No. of issues	No. of documents	%
2000	1	4	16	3
2001	1	4	25	4
2002	1	4	24	4
2003	1	4	33	6
2004	1	4	33	6
2005	1	4	32	5
2006	1	4	33	6
2007	1	4	38	6
2008	1	4	35	6
2009	1	4	32	5
2010	1	4	24	4
2011	1	4	24	4
2012	1	4	33	6
2013	1	4	34	6
2014	1	4	36	6
2015	1	4	36	6
2016	1	5	42	7
2017	1	6	56	10
Total	18	75	586	100

### Document type

In the analysis of the type of records during the studied 18-year period, the absolute majority of the published documents (n = 578) consisted of original articles (n = 562; 97.3%), followed by review articles (n = 12, 2%), and letter to the editor (n = 4, 0.7%). The remaining five were in the form of note (n = 3), conference paper (n = 1), or editorial (n = 1), which were excluded from this analysis.

### Subject areas

The subject areas of the published articles were categorized in four groups. Documents may have more than one subject area. The most common areas for IBJ documents were biochemistry, genetics, and molecular biology (n = 581), followed by medicine (n = 489), immunology, and microbiology (n = 94), as well as material science (n = 94).

### Citation and document trend

**Fig. 1:**
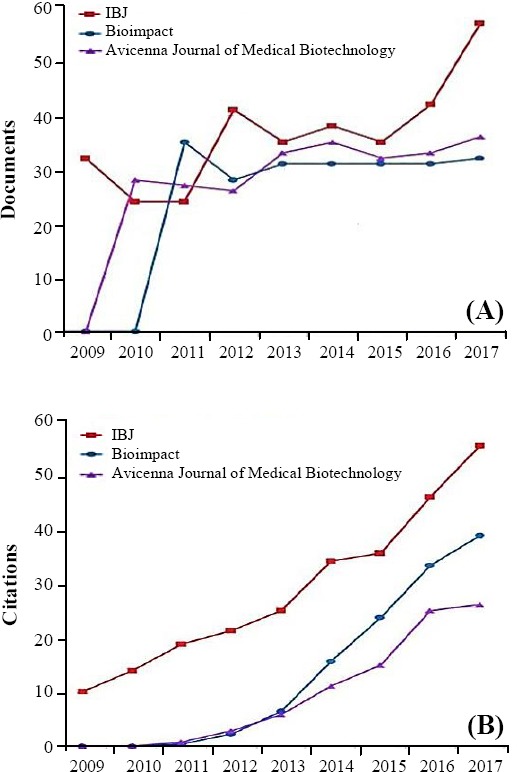
(A) Document and (B) citation trends of (2009-2017) in comparison with Avicenna Journal of Medical Biotechnology and Bioimpact.

We made a comparison between the number of citations/year of IBJ (for the period of 2009-2017) and two other national journals, namely Avicenna Journal of Medical Biotechnology and Bioimpact, founded in 2009 and 2010, respectively. These two journals were selected based on their subject areas and Q, according to the Scimago Database. All three journals are active in the fields of biochemistry, genetics, and molecular biology and are now at levels Q2 and Q3. The three journals possess similar document trends for the mentioned nine-year period, as observed in Figure 1A. However, IBJ holds a much higher citation trend for this time period (Fig. 1B).

### Country territory

**Fig. 2:**
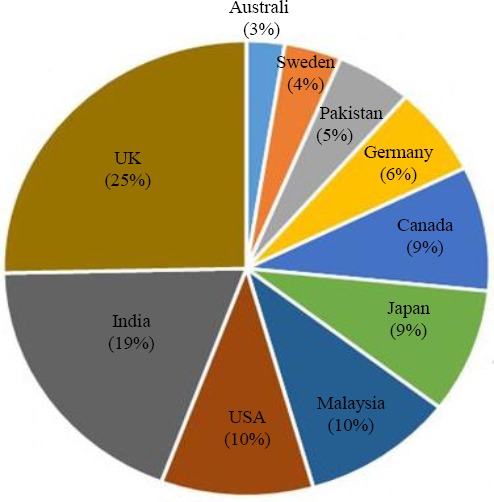
The percentage of documents published in IBJ by country territories other than Iran

Iran constituted 90% of the country territory for the 586 documents of IBJ. The remaining 10% was distributed amongst 10 other countries, most of which belonged to UK (25%; Fig. 2).

### CiteScore

**Fig. 3:**
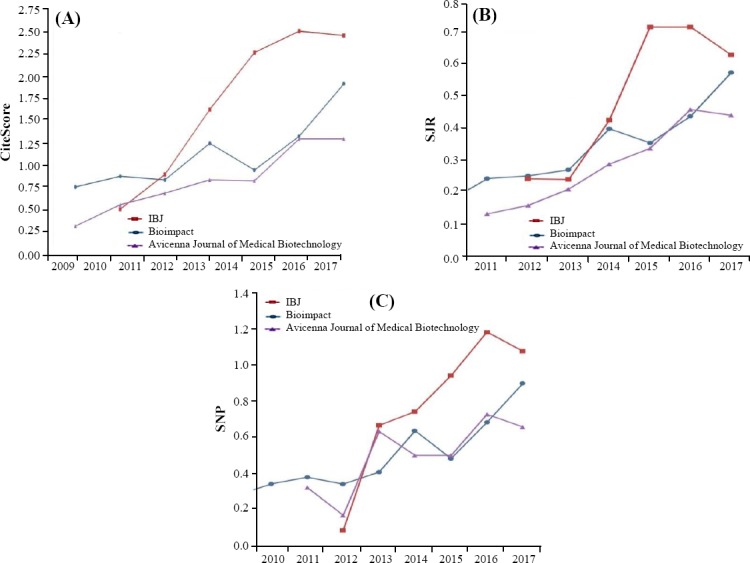
(A) CiteScore, (B) SJR, (C) SNP trends for IBJ (2011-2017).

The CiteScore trend for IBJ for the period of 2011-2017 ranged from 0.76 to 1.92, which peaked in 2017 (1.92; Fig. 3A). The highest trends for journals Avicenna Journal of Medical Biotechnology and Bioimpact 2017 were 1.3 and 2.46, respectively.

### SJR

We assessed the SJR trend for three journals (Fig. 3B). This analysis revealed a significant rise in SJR scores for IBJ, reaching 0.574 in 2017. The highest trends for journals Avicenna Journal of Medical Biotechnology and Bioimpact 2017 were 0.441 and 0.629, respectively.

### SNIP

The analysis of SNIP scores for IBJ (Fig. 3C) revealed an increasing trend of source normalization, peaking in the years 2011 and 2107. The highest trends for the other two Journals in 2017 were 0.735 and 1.99, respectively.

### Q Trends

Q trend analysis for IBJ assigned this journal mainly to Q3-Q4 in most subject areas (Table 2). However, it was able to reach Q2, in the field of biochemistry, genetics, and molecular biology, as well as medical biochemistry since 2016.

**Table 2:** Subject area quartile (Q) of IBJ (2000-2016)

Year	Subject areas		

	Biochemistry, genetics and molecular biology	Biochemistry (medical)	Clinical biochemistry
2000	Q4	Q4	Q4
2001	Q4	Q4	Q4
2002	Q4	Q4	Q4
2003	Q4	Q4	Q4
2004	Q4	Q4	Q4
2005	Q4	Q3	Q4
2006	Q4	Q4	Q4
2007	Q4	Q3	Q4
2008	Q4	Q3	Q4
2009	Q3	Q3	Q4
2010	Q3	Q3	Q4
2011	Q3	Q3	Q3
2012	Q3	Q3	Q3
2013	Q3	Q3	Q3
2014	Q3	Q3	Q3
2015	Q3	Q3	Q3
2016	Q2	Q3	Q3

## RESULTS AND DISCUSSION

The results of this paper can be very helpful for policy makers of IBJ to promote the journal activities. The scientometric analysis, presented here, has evaluated all the published documents in IBJ over a period of 18 years (2000-2017). This study reveals the journal metrics, which can be important for the authors when submitting an article to IBJ.

Scientometric studies evaluated in this paper highlight the bibliometric measures to characterize the article and journal productivity and citation analysis. The article productivity reviews the number of articles and volumes published in a journal in the past years. The journal productivity analyzes the published papers by type, subject area, and active countries in the publication of the article in a journal. The indicators of citation analyses included CiteScore, SJR, and SNIP, which help to evaluate the scientometric trend line of a journal.

Owing to the evaluated scientometric indicators and comparison with the other journals, IBJ is suggested to follow the following comments to improve its level in near future:

-Indexing the Journal in free access databases, such as EBSCOhost and DOAJ. This activity can increase the access to the published articles of IBJ, and also can increase the citation and visibility of the journal.
-Awarding an “Annual Award” to the article that receives the highest citation, without self-citation, after a one-year period of time.
-Informing the audience about the indexing databases and new indicators, such as Altmetrics, on the home page of the journal website to see this information at a glance.
-Focusing and reinforcing on the subject areas of the accepted papers to be published are important, especially in the areas of biochemistry, genetics, and molecular biology, which the journal have capacity to reach to Q1 level in Scimago database.
